# Supplementary material for: Burnout among healthcare providers: Its prevalence and association with anxiety and depression during the COVID-19 pandemic in Macao, China
Source: PLoS One. 2023 Mar 16;18(3):e0283239. doi: 10.1371/journal.pone.0283239 (PMC10019613; doi:10.1371/journal.pone.0283239)
Supplement: Attachment — Submitted filename: Letter of Response.pdf [file pone.0283239.s001.pdf]

Letter of Response

Dear editor,

Thank you for your interest in pursuing our manuscript entitled "Burnout among healthcare providers: Its prevalence and association with anxiety and depression during the COVID-19 pandemic in Macao, China (Manuscript ID: PONE-D-22-29630)" for publication by providing us with an opportunity to improve our manuscript. Please find our responses to the comments raised by the reviewers and the request to rephrase some of the sentences in the following.

Sincerely,
Dr Carolina Ung

Journal Requirements:

When submitting your revision, we need you to address these additional requirements.

1. Please ensure that your manuscript meets PLOS ONE's style requirements, including those for file naming. The PLOS ONE style templates can be found at https://journals.plos.org/plosone/s/file?id=wjVg/PLOSOne_formatting_sample_main_body.pdf and https://journals.plos.org/plosone/s/file?id=ba62/PLOSOne_formatting_sample_title_authors_affiliations.pdf

Response:

Thank you for drawing our attention to the requirements. Revisions to the manuscript have been made accordingly.

2. Thank you for stating the following financial disclosure: "The research was funded by the University of Macau (SRG2021-00007-ICMS)."

Please state what role the funders took in the study. If the funders had no role, please state: "The funders had no role in study design, data collection and analysis, decision to publish, or preparation of the manuscript." If this statement is not correct you must amend it as needed.

Please include this amended Role of Funder statement in your cover letter; we will change the online submission form on your behalf.

Response:

Thank you for drawing our attention to the requirements. Revisions to the cover letter have been made accordingly.

3. In your Data Availability statement, you have not specified where the minimal data set underlying the results described in your manuscript can be found. PLOS defines a study's minimal data set as the underlying data used to reach the conclusions drawn in the manuscript and any additional data required to replicate the reported study findings in their entirety. All PLOS journals require that the minimal data set be made fully available. For more information about our data policy, please see http://journals.plos.org/plosone/s/data-availability.

Upon re-submitting your revised manuscript, please upload your study's minimal underlying data set as either Supporting Information files or to a stable, public repository and include the relevant URLs, DOIs, or accession numbers within your revised cover letter. For a list of acceptable repositories, please see http://journals.plos.org/plosone/s/data-availability#loc-recommended-repositories. Any potentially identifying patient information must be fully anonymized.

Important: If there are ethical or legal restrictions to sharing your data publicly, please explain these restrictions in detail. Please see our guidelines for more information on what we consider unacceptable restrictions to publicly sharing data: http://journals.plos.org/plosone/s/data-availability#loc-unacceptable-data-access-restrictions. Note that it is not acceptable for the authors to be the sole named individuals responsible for ensuring data access.

We will update your Data Availability statement to reflect the information you provide in your cover letter.

Response:

Thank you for drawing our attention to the requirements. Revisions to the cover letter have been made accordingly. The following has been added to the Data Availability section in the submission system:

"Data Availability: The survey data is available on request provided that approval for sharing the anonymised data is granted by the Panel on Research Ethics of the Research Committee, University of Macau. To request such access, please email rskto.ethics@um.edu.mo."

Reviewers' comments:

Reviewer's Responses to Questions

Comments to the Author

1. Is the manuscript technically sound, and do the data support the conclusions?

The manuscript must describe a technically sound piece of scientific research with data that supports the conclusions. Experiments must have been conducted rigorously, with appropriate controls, replication, and sample sizes. The conclusions must be drawn appropriately based on the data presented.

Reviewer #1: Yes

Reviewer #2: Yes

Response:

We appreciate the endorsement from the reviewers.

---

2. Has the statistical analysis been performed appropriately and rigorously?

Reviewer #1: No

Reviewer #2: Yes

Response:

We appreciate the endorsement from Reviewer 2 and will address Reviewer 1's concerns in the following.

---

3. Have the authors made all data underlying the findings in their manuscript fully available?

The PLOS Data policy requires authors to make all data underlying the findings described in their manuscript fully available without restriction, with rare exception (please refer to the Data Availability Statement in the manuscript PDF file). The data should be provided as part of the manuscript or its supporting information, or deposited to a public repository. For example, in addition to summary statistics, the data points behind means, medians and variance measures should be available. If there are

restrictions on publicly sharing data—e.g. participant privacy or use of data from a third party—those must be specified.

Reviewer #1: Yes

Reviewer #2: Yes

Response:

We appreciate the endorsement from the reviewers.

---

4. Is the manuscript presented in an intelligible fashion and written in standard English?

PLOS ONE does not copyedit accepted manuscripts, so the language in submitted articles must be clear, correct, and unambiguous. Any typographical or grammatical errors should be corrected at revision, so please note any specific errors here.

Reviewer #1: Yes

Reviewer #2: Yes

Response:

We appreciate the endorsement from the reviewers.

---

5. Review Comments to the Author

Please use the space provided to explain your answers to the questions above. You may also include additional comments for the author, including concerns about dual publication, research ethics, or publication ethics. (Please upload your review as an attachment if it exceeds 20,000 characters)

Reviewer #1:

1. Introduction section

Burnout has been shown in studies to be common in HPs, and it is closely related to anxiety and depression, according to the authors (line 73-78). Given the high prevalence of burnout in HPs, which has been linked to anxiety and depression, the question here is, what gaps will be addressed in this study, and what makes this study unique? What new body of knowledge has been added to the global community? The rationale What are the gaps that need to be filled, and what makes it unique? This is not convincing and should be demonstrated clearly.

Response:

Thank you for the comments. Major revisions have been made to the Introduction section to highlight the research gaps, the rational of this study, and the new knowledge to be generated from this study findings.

2. Methods sections

2.1 Study design - The study design should be rewritten succinctly —-too much information

Response:

Thank you for the comment. Major revisions have been made to the Methods section accordingly.

2.2 The study design should explain why a survey is the best method of data collection for the study.

Response:

Thank you for the comment. For the purpose of this study, a survey research design allowed data collection to be completed in a relatively short period so that a snapshot of the burnout phenomenon among HPs during the non-acute phase of the COVID-19 pandemic could be depicted in a timely manner amid the continuous progress of the situation. Such information has been added to the first paragraph of the section of Materials and methods.

2.3 The statement regarding ethical consideration is also included in the study design section and is suggested to be separate and placed at the end of the method section.

Response:

Thank you for the comment. The statement regarding ethical consideration has been moved under the subsection of Data collection in the Materials and methods section.

2.4 Study setting and target population - The target populations were all Macao-based HPs. But who exactly are the HPs?

Response:

Thank you for the question. We have added the following information to the subsection of Study target in the Materials and methods section.

"The target population of this study was all the HPs practicing in Macao such as doctors, nurses, pharmacists, medical laboratory technologists, Traditional Chinese Medicine doctors, etc." On the other hand, the professional categories of the participants were also listed in Table 1 of the original manuscript (Table 2 in the revised manuscript).

2.5 In which setting do HPs practice (hospital, health center...???),

Response:

Thank you for the question. Participants' workplace information (e.g. public sector or non-public sector; hospital setting or non-hospital setting) was provided in Table 1 in the original manuscript (Table 2 in the revised manuscript).

2.6 Are healthcare providers registered with the Health Bureau in Macau as of 2020 but not practicing or living in Macau included or excluded from the study??

Response:

Thank you for the question. As stated in subsection of the Questionnaire design in the Materials and methods section in the original manuscript, the first question in the questionnaire was for the respondents to confirm whether they were HPs practicing in Macao. Only those who had confirmed their place of practice being in Macao were included in this study.

In addition, in order to improve clarify, "All respondents confirmed that their place of practice was in Macao." has been added to the second paragraph of the Results section.

2.7 All this suggest that the target population be clearly defined by clearly indicating who is included and who is excluded.

Response:
Thank you for the comment. We hope that above responses would be deemed sufficient to address the concern.

2.8 Sampling methods and size - The author simply stated 371 as a minimum sample size, but the sampling techniques used to select the sample are unclear —- suggest that the sampling methods used to select these observational units be described.

Response:
Thank you for the comment. Convenience and snowballing sampling method was employed in this study. Such information has been added to the subsection of Data collection in the Materials and methods section.

2.9 Furthermore, a contradictory statement is written at the beginning of the methods sections: "open and online survey to be completed voluntarily by healthcare providers in Macao between October and December 2021." (line 102) Respondents who had completed the COVID-19 duties were invited to complete the Section 4 questions. This section was created to investigate HP's perception of health

workforce management during the COVID-19 pandemic and to identify areas where improvements could help to mitigate burnout (line 192-194)

- Are these study participants distinct from the 371?
- How many of these took part?
- How were these participants chosen to respond to Section 4?

Response:

Thank you for the question. The following clarification is provided for your kind re-consideration. As stated in the original manuscript,

- According to Line 132-133, "371" was only referring to the minimum sample size calculated for the current study (confidence level 95%, margin of error 5%).
- According to Line 261-264, "A total of 622 people visited the survey link, of which 616 gave consent to participate, giving a participation rate of 99%. Out of the 616 respondents who agreed to participate and attempted the survey, 498 surveys were completed, giving a completion rate of 80.8%." Indeed, 498 respondents were included in this study (Section 1 to 3).
- As reported in Table 1 in the original manuscript (Table 2 in the revised manuscript), among the 498 respondents, 413 had been involved in COVID-19 related professional duties
- According to Line 331, "Among 413 respondents who had participated in the COVID-19 related duties, 396 opted to continue into Section 4 of the questionnaire.."

We hope that the clarification above would be deemed sufficient to address the concern.

2.10 The author gathers data through volunteerism (volunteer sampling) and snowballs sampling. These approaches, commonly known as convenience sampling, have the lowest credibility of any sampling method and should only be used as a last resort.

Response:

Thank you for the comment. We agree that selection bias and response bias are inevitably given the nature of the data collection approaches. In the Limitations section of the original manuscript, we acknowledged such shortcomings. In the revised manuscript, we have also added "Bias towards potential volunteerism might have also inevitably increased sampling error, affecting the generalizability of the study findings and restricting the inference made about the entirety of the HP population." to emphasize the impact of such shortcomings.

2.11 Analysis - the reason /assumption why using Spearman's rho test are not justified

Response:

Thank you for the comment. We would like to clarify that Spearman's rho was used in Table 5 as shown in the original script considering that as Spearman's rho is recommended for reporting correlations related to Likert scale items and a non-parametric measure of rank correlation could not be ruled out. We have revised the subsection of Data analysis accordingly to better indicate this.

3. Result

3.1 The number of study participants reported in the results section is inconsistent with the calculated sample size (377) vs actual participation (498).

Response:

Thank you for the question. The following clarification is provided for your kind re-consideration. As stated in the original manuscript,

- According to Line 132-133, "371" was only referring to the minimum sample size calculated for the current study (confidence level 95%, margin of error 5%).
- According to Line 261-264, "A total of 622 people visited the survey link, of which 616 gave consent to participate, giving a participation rate of 99%. Out of the 616 respondents who agreed to participate and attempted the survey, 498 surveys were completed, giving a completion rate of 80.8%." Indeed, 498 respondents were included in this study (Section 1 to 3).

We hope that the clarification above would be deemed sufficient to address the concern.

3.2 Logistic regression analysis - There is no evidence that the authors checked logistic regression assumptions (such as the assumption of collinear relationships among explanatory variables-correlation of |r|, VIF value, etc.) before using the fitted model to infer the relationship between the response variable and the explanatory variables.

Response:

Thank you for the comment. Before we ran the logistic regression analysis, an analysis of standard residuals was carried out, which showed that the data contained no outliers considering standard residual minimum = -2.435, standard residual maximum = 2.215 for personal burnout; standard residual minimum = -2.241, standard residual maximum = 2.087 for work burnout; and standard residual minimum = -1.75, standard residual maximum = 2.214 for personal burnout (standardized residuals beyond ± 3 are usually considered to have outliers).

Residuals Statistics					
	Minimum	Maximum	Mean	Std. Deviation	N
Predicted Value	-0.0225	1.177	0.604	0.284	498.000
Residual	-0.98585	0.860	0.000	0.399	498.000
Std. Predicted Value	-2.207	2.015	0.000	1.000	498.000
Std. Residual	-2.435	2.125	0.000	0.985	498.000
a Dependent Variable: Personal burnout					

Residuals Statistics					
	Minimum	Maximum	Mean	Std. Deviation	N
Predicted Value	-0.1368	1.094	0.506	0.263	498.000
Residual	-0.96909	0.902	0.000	0.426	498.000
Std. Predicted Value	-2.445	2.236	0.000	1.000	498.000
Std. Residual	-2.241	2.087	0.000	0.985	498.000
a Dependent Variable: Work burnout					

Residuals Statistics					
	Minimum	Maximum	Mean	Std. Deviation	N
Predicted Value	-0.2186	0.822	0.315	0.207	498.000
Residual	-0.74044	0.937	0.000	0.417	498.000
Std. Predicted Value	-2.585	2.455	0.000	1.000	498.000
Std. Residual	-1.75	2.214	0.000	0.985	498.000
a Dependent Variable: Client burnout					

On the other hand, tests conducted to check if the data met the assumption of collinearity showed that multicollinearity was not a concern considering tolerance >0.1 and variance inflation factor (VIF) <5 for all 15 independent variables (considering that only VIF greater than 5 or 10 are usually suggested for detecting multicollinearity). More specifically, all the VIF ranged from 1-2.5 except for age (VIF 4.814) and years of practice (VIF 4.600). The slightly higher VIF for age and years of practice is understandable considering that, in general, the older the participant was, the longer he or she would have practiced.

Coefficients								
Model		Unstandardized Coefficients		Standardized Coefficie	t	Sig.	Collinearity Statistics	
		B	Std. Error	Beta			Tolerance	VIF
1	(Constant)	0.231	0.314		0.735	0.463		
	Age (years)	-0.065	0.057	-0.091	-1.150	0.251	0.208	4.814
	Gender	0.022	0.057	0.014	0.385	0.701	0.979	1.022
	Marital status	0.020	0.073	0.014	0.276	0.783	0.470	2.127
	Being a parent	0.079	0.093	0.048	0.849	0.397	0.411	2.433
	Highest Education level	0.037	0.046	0.030	0.805	0.421	0.920	1.086
	Hisoty of chronic diseases or severe illnesses	0.109	0.081	0.052	1.340	0.181	0.845	1.184
	Professional category	-0.007	0.010	-0.028	-0.734	0.463	0.862	1.161
	Years of practice	-0.105	0.062	-0.131	-1.696	0.091	0.217	4.600
	Professional seniority	0.024	0.021	0.045	1.135	0.257	0.830	1.206
	Management duty	0.130	0.072	0.073	1.818	0.070	0.802	1.246
	Workplace_Public	0.219	0.070	0.132	3.133	0.002	0.725	1.379
	Workplace_Hospital	0.065	0.069	0.039	0.943	0.346	0.764	1.310
	Professional experience with COVID-19	0.017	0.087	0.008	0.196	0.844	0.829	1.207
	Anxiety status	0.424	0.051	0.389	8.270	<.001	0.583	1.716
	Depression status	0.238	0.046	0.241	5.171	<.001	0.595	1.680
a Dependent Variable: Personal burnout								

Coefficients								
Model		Unstandardized Coefficients		Standardized Coefficie	t	Sig.	Collinearity Statistics	
		B	Std. Error	Beta			Tolerance	VIF
1	(Constant)	0.037	0.269		0.136	0.892		
	Age (years)	-0.052	0.049	-0.088	-1.062	0.289	0.208	4.814
	Gender	0.092	0.049	0.072	1.895	0.059	0.979	1.022
	Marital status	0.068	0.063	0.059	1.085	0.278	0.470	2.127
	Being a parent	0.035	0.080	0.026	0.442	0.659	0.411	2.433
	Highest Education level	-0.015	0.039	-0.015	-0.385	0.701	0.920	1.086
	Hisoty of chronic diseases or severe illnesses	-0.003	0.069	-0.002	-0.048	0.962	0.845	1.184
	Professional category	0.001	0.008	0.004	0.093	0.926	0.862	1.161
	Years of practice	-0.060	0.053	-0.091	-1.124	0.261	0.217	4.600
	Professional seniority	0.017	0.018	0.039	0.949	0.343	0.830	1.206
	Management duty	-0.005	0.061	-0.003	-0.074	0.941	0.802	1.246
	Workplace_Public	0.018	0.060	0.013	0.298	0.766	0.725	1.379
	Workplace_Hospital	0.190	0.059	0.138	3.208	0.001	0.764	1.310
	Professional experience with COVID-19	0.067	0.074	0.037	0.901	0.368	0.829	1.207
	Anxiety status	0.333	0.044	0.374	7.593	<.001	0.583	1.716
	Depression status	0.120	0.039	0.148	3.038	0.003	0.595	1.680
a Dependent Variable: Work burnout								

Coefficients								
Model		Unstandardized Coefficients		Standardized Coefficie	t	Sig.	Collinearity Statistics	
		B	Std. Error	Beta			Tolerance	VIF
1	(Constant)	-0.161	0.273		-0.589	0.556		
	Age (years)	0.025	0.049	0.045	0.509	0.611	0.208	4.814
	Gender	0.085	0.049	0.070	1.722	0.086	0.979	1.022
	Marital status	0.047	0.064	0.043	0.733	0.464	0.470	2.127
	Being a parent	-0.013	0.081	-0.010	-0.166	0.868	0.411	2.433
	Highest Education level	-0.051	0.040	-0.054	-1.296	0.196	0.920	1.086
	Hisoty of chronic diseases or severe illnesses	0.047	0.070	0.029	0.671	0.503	0.845	1.184
	Professional category	-0.005	0.008	-0.024	-0.555	0.579	0.862	1.161
	Years of practice	-0.130	0.054	-0.208	-2.404	0.017	0.217	4.600
	Professional seniority	0.015	0.018	0.036	0.804	0.422	0.830	1.206
	Management duty	-0.065	0.062	-0.047	-1.037	0.300	0.802	1.246
	Workplace_Public	0.048	0.061	0.037	0.789	0.431	0.725	1.379
	Workplace_Hospital	0.051	0.060	0.039	0.847	0.397	0.764	1.310
	Professional experience with COVID-19	0.158	0.075	0.093	2.100	0.036	0.829	1.207
	Anxiety status	0.222	0.045	0.262	4.972	<.001	0.583	1.716
	Depression status	0.109	0.040	0.143	2.730	0.007	0.595	1.680
a Dependent Variable: Client burnout								

To improve clarity, we have added the following to the subsection of Logistic regression analysis:

"An analysis of standard residual (SR) showed that the data about burnout contained no outliers as none of the SRs were beyond ±3 (SR minimum=-2.435, SR maximum= 2.215 for personal burnout; SR minimum=-2.241, SR maximum=2.087 for work-related burnout; SR minimum=-1.75, SR maximum=2.214 for client-related burnout). Tests were also conducted to show multicollinearity was not a concern for the 15 variables (including demographic and professional characteristics, as well as anxiety and depression status) considering that tolerance for all the variables was greater than 0.1 and none of the variance inflation factor (VIF) was greater than 5."

3.3 Furthermore, the reported OR is unclear as to whether it is log odds or adjusted (exponatiated);

Response:

Thank you for the comment. The OR shown in Table 5 was the unadjusted odds ratios using a reference category. To improve clarity, the following has been added to the manuscript.

"The unadjusted odds ratios indicated that: (1) HPs in the public sector were 2.088 (95% CI 1.160-3.758) times more likely to experience moderate to severe personal burnout compared to HPs in non-public sector; (2) HPs in the hospital setting were 2.280 (95% CI 1.319-3.943) times more likely to experience moderate to severe work-related burnout compared to HPs in non-hospital setting; and (3) HPs who undertook COVID-19 professional duties were 2.421 (95% CI 1.218-4.811) times more likely to experience moderate to severe client-related burnout compared to HPs who did not." and "In addition, HPs with abnormal cases of anxiety were 14.326 times (95% CI 4.317-47.543), 8.169 times (95% CI 3.662-18.224) and 3.211 times (95% CI 1.525-6.762) more like to experience moderate to severe personal, work-related and client-related burnout respectively when compared to HPs with no anxiety. Similarly, HPs with abnormal cases of depression were 12.050 times (95% CI 5.420-26.791), 3.965 times (95% CI 2.034-7.729) and 4.180 times (95% CI 2.074-8.423) more like to experience moderate to severe personal, work-related and client-related burnout respectively when compared to HPs with no depression."

3.4 The reference category of the variables entered into the model is unknown;

Response:

Thank you for the comment. The reference category for each independent variables has been added to Table 5.

3.5 Logistic regression analysis, such as OR and CI, is not interpreted.

Response:

Thank you for the comment. As responded above to comment 3.3, additional interpretation has been added to the manuscript.

3.6 the direction and the strength of correlation is not mentioned

Response:

Thank you for the comment and we agree that we can further interpret the direction and the strength of correlation reported in Table 6. As such, we have added the following to the subsection of HPs' experiences with COVID-19 related duties:

"Spearman's correlation was computed to assess the relationship between the level of agreements on the experiences with COVID-19 related duties to the likelihood of moderate to severe personal, work-related, and client-related burnout. Apart from statements 1 and 3, all the other statements were negatively associated with burnout. The positive values of Spearman's rho in Statements 1 (r(df) = 0.275, $p < 0.01$; r(df) = 0.259, $p<0.01$; r(df) = 0.257, $p<0.01$) and Statement 3 (r(df) = 0.145, $p<0.05$; r(df) = 0.133, $p<0.05$; r(df) = 0.201, $p<0.01$) indicated that the more the respondents perceived a risk of infection with COVID-19 at workplace or had the need for psychological support, the more likely they were going to experience different types of moderate to severe burnout" and "On the contrary, the negative values of Spearman's rho in all the remaining statements which described different sources of support, sound management practice and perceived health status indicated that all these factors were negatively associated with the likelihood of burnout. According to the results of Statement 14, high level of perceived health (r(df) = -0.488, $p < 0.01$; r(df) = -0.417, $p<0.01$; r(df) = -0.411, $p<0.01$) had the strongest negative association with moderate to severe burnout. Other important factors such as Statement 13 - being able to rest and recover during breaks when participating in COVID-19 related duties (r(df) = -0.384, $p<0.01$), Statement 11 - a sense of job satisfaction when participating in COVID-19 related duties (r(df) = -0.455, $p<0.01$), and Statement 9 – supportive superior (r(df) = -0.378, $p<0.01$) also showed significant negative association with moderate to severe personal, work-related, and client-related burnout respectively."

4. Discussion

4.1 Suggest to Start by restating the research question, followed by discussing the main findings and then interpretation/explain its implication(result (explaining the result –based on theories or researchers logical analysis .

Response:

Thank you for the comment. Major revisions to the Discussion section have been made accordingly.

4.2 Compare findings in light of other, similar studies' findings. If difference, explain the differences (methods, setting, participants)

Response:

Thank you for the comment. Major revisions to the Discussion section have been made accordingly.

4.3 Indicate Benefits or implications of the findings for patients, practitioners, health care organizations, researchers, or policymakers?

Response:

Thank you for the comment. Major revisions to the Discussion section have been made accordingly.

4.4 State clearly what your study added to the research area and potential further research that should be conducted. (future areas of research)

Response:

Thank you for the comment. Major revisions to the Discussion section have been made accordingly.

4.5 Strength and limitation

Response:

Thank you for the comment. Major revisions to the Discussion section have been made accordingly.

4.6 Conclusion and Recommendation

Response:

Thank you for the comment. Major revisions to the Discussion section have been made accordingly.

Reviewer #2:

1. Overall, this is an excellent article and very well written. The impact of COVID-19 on health and care workers is possibly one of the key public health issues of our time, with potential consequences that can reverberate for the next few years and decades. I would like to congratulate the authors for such a well developed and comprehensive paper that examines burnout and various determinant of burnout among HPs.

Response:

Thank you for the encouraging comment.

2. I only have two suggestions for the consideration of the authors:

2.1 In the Introduction, it would be good to highlight that the COVID-19 pandemic has had tremendous impacts on the physical and mental health and wellbeing of the HPs as well as on their distribution and on their working conditions. This multidimensional impact is quite extraordinary, and there is an interplay of factors which often exacerbate each other. The WHO Weekly Epidemiological Update on COVID-19 published on 30 March 2021 (https://www.who.int/publications/m/item/weekly-epidemiological-update-on-covid-19---31-march-2021) first revealed this comprehensive framework for measuring the impact of COVID-19 on HPs and this has since been tested and validated across countries of various settings (https://www.who.int/publications/i/item/9789240039001; https://www.who.int/publications/m/item/impact-of-covid-19-on-human-resources-for-health-and-policy-response--the-case-of-belize--grenada--and-jamaica). Burnout has been highlighted as one of the impacts in the mental health dimension of the framework. So it would be ideal to include a citation for the multidimensional framework, just to sensitize the readers that burnout is just one of the multitudes of impacts of the pandemic on HPs.

Response:

Thank you for the encouraging comment. We have added this important information and quoted the WHO document in the Introduction section.

2.2 A recent publication has looked at the mental health impact of COVID-19 on HPs. It highlights that burnout has been an issue for HPs even before the pandemic, but COVID-19 has exacerbated this further (pages 12 and 13). Section 2 presents interventions to protect the mental health of HPs at the organizational level and psychosocial interventions, while Section 3 presents policy actions and recommendations, which are very much in line with the interventions and recommendations outlined in this paper. Hence, I would suggest that you reference this report at the very least, and if needed, tweak language to be more aligned with it. The report is titled "Our duty of care: A global call to action to protect the mental health of health and care workers" and can be accessed here - https://2022.wish.org.qa/wp-content/uploads/2022/10/QFJ9259-02-Our-Duty-Of-Care-WEB.pdf

Response:

Thank you for the encouraging comment. We have referred to some of the recommendations proposed in the document and quoted the document in the Introduction section as well as in the Discussion section [reference 18].

6. PLOS authors have the option to publish the peer review history of their article (what does this mean?). If published, this will include your full peer review and any attached files.

If you choose "no", your identity will remain anonymous but your review may still be made public.

Response:

Thank you. Publishing the peer review history of their article is fine with the authors.

Do you want your identity to be public for this peer review? For information about this choice, including consent withdrawal, please see our Privacy Policy.

Reviewer #1: No

Reviewer #2: No
